# The GHKL ATPase Family as a Paradigm for MutL Homolog Function in DNA Mismatch Repair

**DOI:** 10.3390/ijms262412157

**Published:** 2025-12-18

**Authors:** Jonathan M. Piscitelli, Carol M. Manhart

**Affiliations:** Department of Chemistry, Temple University, Philadelphia, PA 19122, USA; tuo69519@temple.edu

**Keywords:** DNA mismatch repair, DNA repair, ATPases, MutL, GHKL

## Abstract

ATP hydrolysis drives essential processes across biology, from nucleic acid translocation and conformational switching to signal transduction. The GHKL ATPase family—DNA Gyrase B, Heat Shock Protein 90 (Hsp90), Histidine Kinases, and MutL homologs—shares a Bergerat-fold that couples nucleotide binding and hydrolysis to conformational changes, dimerization, and signaling. Despite their diverse roles, GHKL proteins rely on common ATP-dependent principles. Within this family, MutLα (MLH1-PMS2 in humans, Mlh1-Pms1 in yeast) is central to eukaryotic mismatch repair, where it provides the endonuclease activity needed for strand incision and coordinates interactions with other repair partners. MutLα exemplifies how the Bergerat-fold has been adapted to regulate DNA interactions, partner communication, and protein turnover on DNA. By examining MutLα through the lens of other GHKL proteins, we can clarify how ATP binding and hydrolysis drive its conformational dynamics, nuclease activation, and regulation within its pathway, highlighting how conserved mechanistic strategies are repurposed across biological systems.

## 1. Enigmatic Features of MutL Homolog ATPase Activity

Mismatch repair (MMR) is a conserved pathway that preserves genome stability by correcting replication errors, a process that depends on MutL proteins and their ATPase activity [1,2,3,4,5,6,7,8,9]. Mutation studies have long established that loss of this activity compromises MMR, yet how MutL proteins functionally use ATP binding and hydrolysis in repair remains unresolved. Unlike helicases or polymerases, which use ATP as fuel for processive motion, MutL’s ATPase activity appears to act primarily as a molecular signal, but how this signal licenses MutL’s activities is not well understood. MutL belongs to a broader ATPase family whose shared mechanisms may provide insight into how it uses ATP in MMR.

The MMR pathway prevents the accumulation of mutations that could compromise genome integrity, by removing and resynthesizing mispaired nucleotides that escape polymerase proofreading. The pathway begins when a MutS homolog scans DNA for mispaired bases and, upon recognition, recruits a MutL homolog to direct downstream processing [10]. In *Escherichia coli*, MutL acts as a molecular matchmaker, linking MutS to the endonuclease MutH, which incises hemimethylated GATC sites on the newly synthesized strand. In contrast, many bacteria, including *Bacillus subtilis*, and all eukaryotes lack MutH and instead rely on MutL homologs with intrinsic endonuclease activity to introduce nicks into the nascent strand, which may already contain discontinuities to potentially serve as strand discrimination marks [11,12,13,14,15,16]. In all cases, the nick generated by MutH or MutL provides an entry point for mismatch excision and subsequent resynthesis by a DNA polymerase [10,11,17,18].

**Figure 1:**
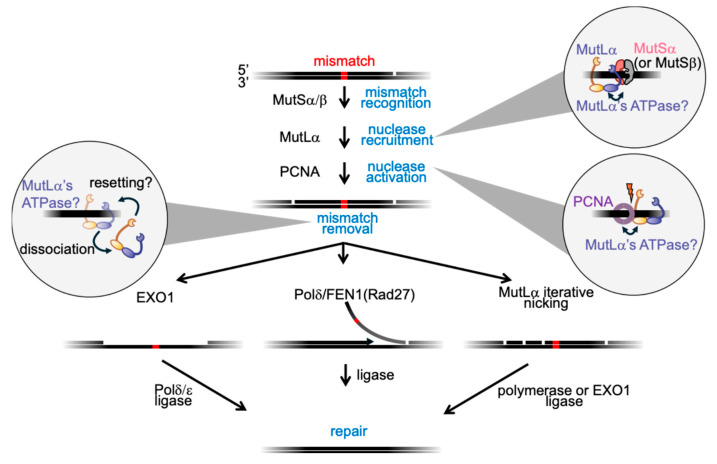
Eukaryotic mismatch repair and potential roles for MutLα’s ATPase activity. Mismatch recognition is carried out by MutSα (primarily base-base mismatches and small insertion/deletion loops) and MutSβ (larger insertion/deletion mismatches, with some overlapping specificity) [29]. Both recruit MutLα (blue and yellow complex) to the mismatch site. MutSα/β (pink and grey complex) are known to bind ATP for recruitment. Whether ATP binding by MutLα is required immediately after its recruitment or instead modulates subsequent MutLα-MutS interactions (double-headed arrow), remains unclear. MutLα’s ATPase activity is stimulated by DNA and PCNA (purple), suggesting potential functions in licensing or positioning endonuclease activity (indicated by lightning bolt), coordinating interactions with repair factors, or enabling protein turnover and multiple rounds of nicking. While this schematic highlights the eukaryotic pathway, analogous ATP-regulated MutL mechanisms may also operate in bacteria that lack MutH and methyl-directed strand discrimination.

Mismatch excision in *E. coli* is carried out by exonucleases (ExoI, VII, X, or RecJ) and the UvrD helicase [19,20,21]. In eukaryotes, mismatch removal has been proposed to occur through at least three pathways, including (i) excision by EXO1 followed by gap filling by Polδ or Polε; (ii) strand displacement synthesis by Polδ coupled with flap cleavage by a flap endonuclease (FEN1 in humans, Rad27 in yeast); or (iii) iterative nicking by MutLα (a heterodimer of the MutL homologs MLH1 with PMS2 in humans, or Mlh1 with Pms1 in yeast) followed by oligonucleotide removal through strand displacement synthesis or excision by EXO1 [10,22,23] (Figure 1).

A central unresolved question in MMR is how MutL and MutLα’s essential, but not well understood, ATPase activity contributes to repair events. Biochemical studies have shown that nucleolytic *B. subtilis* MutL and eukaryotic MutLα can nick DNA nonspecifically in vitro even without ATPase activity [24,25,26], whereas in vivo, MutLα ATPase activity is indispensable [5,6,7,8,9]. This discrepancy suggests that ATP hydrolysis may not be strictly required to catalyze the incision itself but could play multiple roles in the repair context. For example, ATP could play a role in stabilizing MutL/MutLα’s association with DNA or partner proteins, licensing or positioning its endonuclease activity at the correct strand or site, and/or enabling recycling and reset to permit multiple rounds of nicking for mismatch removal.

Insight may come from its membership in the GHKL ATPase family, named for DNA Gyrase B, Heat Shock Protein 90 (Hsp90), Histidine Kinases, and MutL, whose members share a conserved ATP-binding domain structure [27,28]. Across these otherwise diverse proteins, ATP binding and hydrolysis are consistently used to orchestrate conformational switching, dimerization, and pathway signaling. Viewing MutLα through this broader GHKL paradigm provides a framework for identifying the general principles by which ATP hydrolysis drives its roles in eukaryotic mismatch repair.

### 1.1. MutL Homolog Architecture and ATPase-Driven Conformational Changes

**Figure 2:**
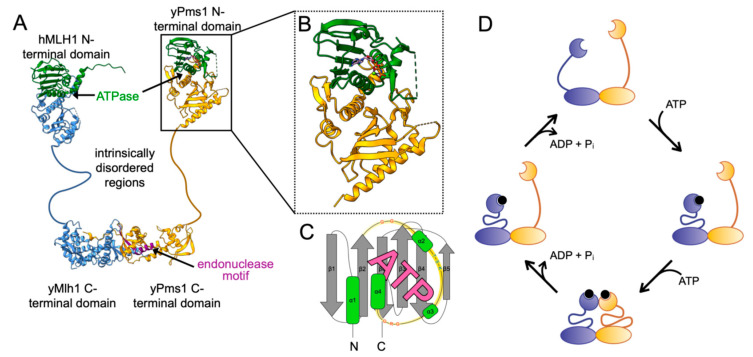
MutLα domain architecture and conserved ATP-binding fold. (**A**) Composite full-length model of the MutLα heterodimer assembled from available crystal structures. The human MLH1 N-terminal domain (blue; PDB: 4P7A) is connected by an intrinsically disordered region to the dimeric C-terminal domains of yeast Mlh1 (blue)-Pms1 (yellow) (PDB: 4E4W). The yeast Pms1 N-terminal domain (yellow; PDB: 3H4L) is similarly positioned and connected to its C-terminal domain by an intrinsically disordered region. This composite highlights the conserved organization of N-terminal ATPase domains flexibly linked to constitutively dimerized C-terminal domains. The Bergerat-fold ATPase site is shown in green and the endonuclease motif in purple in the C-terminal domain of yeast Pms1. (**B**) Magnified view of the structure of the yeast Pms1 N-terminal domain showing the conserved Bergerat-fold (green) bound to an ATP analog (phosphoaminophosphonic acid–adenylate ester). (**C**) Schematic of the Bergerat-fold (adapted from [27] as found in *E. coli* MutL with conserved glycine residues indicated (red), other conserved residues are indicated including a catalytic residue (blue). The ATP “lid” is highlighted in yellow. The amino (N-) and carboxy (C-) termini are indicated. (**D**) Model for the ATPase cycle of MutLα, illustrating asymmetrical usage of ATP (black circles) by the two subunits, domain dimerization, and compaction mediated by intrinsically disordered regions upon ATP binding. The Mlh1/MLH1 subunit is in blue and the Pms1/PMS2 subunit in yellow. Adapted from models in [40,45].

The MMR pathway and MutL proteins are largely conserved across biology. Members of the MutL family function primarily as dimers, forming homodimers in bacteria and, in eukaryotes, heterodimers in which MLH1 pairs with PMS2 (in mammals) or Pms1 (in yeast) as defined above to form MutLα, with PMS1 (in mammals) or Mlh2 (in yeast) to form MutLβ, or with MLH3 to form MutLγ. The nomenclature can be confusing: human PMS2 is orthologous to yeast Pms1, while human PMS1 is orthologous to yeast Mlh2. Among these complexes, MutLα is the major contributor to eukaryotic MMR [2,10,30,31,32,33,34,35]. Across organisms, each MutL homolog subunit contains a globular amino (N)-terminal domain and a globular carboxy (C)-terminal domain connected by an intrinsically disordered linker (Figure 2A). The C-terminal domains are believed to be constitutively dimerized in the absence of nucleotide, forming a stable dimer [2,24,36,37,38,39,40,41,42]. In contrast, the N-terminal domains are separated in the *apo* state but dimerize upon ATP binding, a conformational switch thought to be essential for MMR initiation [4,5,39]. These N-terminal regions contain a conserved ATP-binding pocket amongst the GHKL family called the Bergerat-fold, which features a dynamic “lid” region (Figure 2A–C). The structure of this fold is unique to the GHKL family, distinguishing these ATPases from other major classes such as ABC ATPases, AAA ATPases, and AAA+ ATPases. Mutations to this fold in either subunit of MutLα disrupt conformational changes and elevate mutation rates, underscoring the critical role of ATPase activity and this signature fold [4,5,6,43,44].

Because MutL and MutLα’s functions require ATPase activity in vivo, multiple experimental approaches have probed how ATP binding stabilizes and reorganizes the protein. The role of ATP in MutL stabilization was first shown by crystallography in *E. coli*, where fragments of MutL could only be crystallized in the presence of nonhydrolyzable ADP-PnP but not in the *apo* form, indicating nucleotide binding may be required to order the ATPase domains [40,46]. Proteolysis assays reinforced this hypothesis, where ATP binding stabilized MutL proteins, in *Saccharomyces cerevisiae*, *Thermus thermophilus*, and humans against cleavage, which is consistent with ATP-induced compaction [5,45,47,48].

Global rearrangements in MutL proteins were also observed in the presence of ATP. Full-length *E. coli* MutL eluted later from size-exclusion columns when nucleotide-bound than when in the *apo* form, which may be consistent with compaction into a clamp-like state [39,49]. Evidence of ATP-dependent compaction or clamp closure is also supported by biochemical, structural, and single molecule data [49,50]. Additionally, using single molecule atomic force microscopy experiments, MutL homologs were directly visualized in open, one “arm” closed states (where one subunit was compacted), and semi- and fully condensed states, with ATP binding shifting the equilibrium toward closed, condensed conformations [45,51] (Figure 2D).

Genetic and mechanistic evidence links these structural transitions to repair fidelity. In *E. coli*, introduction of an ATP-binding-deficient mutation prevented clamp closure and resulted in elevated mutagenesis [2]. In yeast, deletion of linker segments thought to mediate clamp opening and closing likewise reduced ATPase activity, impaired nuclease cycling, and resulted in increased mutation rates [25,52]. The functional significance of these transitions was tested more directly in engineered yeast MutLα complexes with chemically inducible dimerization domains. When artificially “locked” in a closed state, the complexes displayed elevated ATPase and nuclease activity, whereas variants unable to close showed nuclease defects. Despite opposite effects on biochemical activity, both perturbations led to increased mutagenesis in vivo, highlighting that regulated cycles of clamp opening and closure are critical for MMR [53].

A recurring feature of MutL is its asymmetric use of ATP. Even though bacterial MutL proteins are homodimers, the two sites appear nonequivalent: one protomer tends to stabilize the closed clamp, while the other hydrolyzes ATP more rapidly [2,5,39,40]. This asymmetry may act as a timing mechanism, ensuring that ATP binding signals clamp closure while hydrolysis in only one subunit is sufficient to destabilize the complex. In eukaryotes, the asymmetry is even more pronounced. Using yeast MutLα, it has been demonstrated that Mlh1 binds ATP with higher affinity and primarily drives clamp formation, whereas Pms1 has faster turnover (Figure 2D). In vivo, this division of labor has clear consequences because mutations impairing ATP binding in Mlh1 sharply increase mutation rates, while equivalent mutations in yeast Pms1 have more modest effects, demonstrating that the two subunits are not interchangeable [5].

Together, these findings suggest that ATP binding and hydrolysis regulate MutL and MutLα’s clamp-like transitions and thereby influence repair efficiency. However, the precise relationship between these conformational changes and specific repair steps remains unclear. It is not clear whether ATP primarily licenses a productive repair state, resets the clamp after engagement, or balances both functions.

### 1.2. Higher-Order MutL Assemblies and Partner Interactions

Beyond the dimeric clamp, MutL homologs can form higher-order assemblies. In *E. coli*, size-exclusion chromatography revealed ~300 kDa species in the absence of ATP, consistent with multimers, while addition of nonhydrolyzable ATP analogs shifted the protein to ~139 kDa dimers [40,54,55]. Crosslinking experiments similarly confirm *apo* multimers, while ADP-PnP stabilizes dimers [39]. DNA binding assays reveal intrinsic ATP-independent cooperative behavior. Both *E. coli* MutL and *S. cerevisiae* MutLα bind longer DNA with higher apparent affinity and sigmoidal isotherms [56,57]. Sucrose density gradient sedimentation analysis also suggests that there is a surplus of human MutLα relative to the mismatch recognition protein MutSα on mismatched DNA [58]. Functionally, MutLα and MutLγ show increased endonuclease activity on longer substrates over shorter DNA [59,60]. Direct visualization by atomic force and electron microscopy experiments corroborate this model, with multiple MutLα/MutLγ particles binding to a single DNA molecule in yeast and human systems, consistent with cooperative assembly [57,60,61].

Although ATP clearly stabilizes dimerization, its influence on DNA binding appears more nuanced. For yeast MutLα, ATP marginally reduces affinity for DNA, suggesting that its primary role may be to regulate conformational state and partner interactions rather than DNA engagement itself [25,26]. DNA binding assays with *E. coli* MutL show somewhat increased affinity in the presence of nonhydrolyzable ATP analogs, consistent with nucleotide-driven stabilization of its DNA-bound state, with hydrolysis potentially releasing the protein from DNA [40]. For both bacterial MutL and eukaryotic MutLα, DNA binding maps to the N-terminal domains, which also harbor the ATPase sites, highlighting a possible close physical and functional coupling between nucleotide state and DNA interactions [40,41,62].

Consistent with this, MutL proteins participate in higher-order complexes with other MMR factors. Interactions with MutS proteins appear to be nucleotide-dependent but do not strictly require MutL’s intrinsic ATPase activity; both ATP binding and hydrolysis-deficient variants retain capacity to associate with MutS complexes, though these interactions may be less efficient in some cases [8,29,34,43,48,54,55,63,64,65,66,67,68,69]. These data do not resolve whether MutL proteins undergo an ATP-dependent step immediately after recruitment to DNA by MutS proteins, a possibility supported by the observation that DNA itself stimulates the ATPase activity of *E. coli* MutL and human and yeast MutLα [25,40,70]. In contrast, interactions with MutH and processivity clamps are more directly tied to MutL’s nucleotide state. In *E. coli*, MutL’s ATPase activity stimulates MutH, while in yeast and human systems, MutLα’s endonuclease activity is largely inactive until stimulated by PCNA, which also enhances MutLα’s ATPase activity [13,14,15,25,26,64,70]. In *B. subtilis*, MutL functions are similarly regulated by the β clamp [2,71,72].

Together, these observations suggest that ATP not only governs MutL’s clamp dynamics but also modulates its interactions with key partners such as MutH, and PCNA. What remains unresolved is how these roles are integrated on DNA: does ATP binding primarily regulate MutL’s interactions with partner proteins, or do partner proteins reshape MutL’s ATPase cycle to control when and where the protein acts along a DNA contour? Framing MutL’s ATPase activity in the broader context of the GHKL family, where ATP consistently acts as a molecular switch to regulate complex processes, may help clarify how these multiple functions coordinate in MMR.

## 2. ATP Hydrolysis and the GHKL ATPase Paradigm

The questions surrounding how ATP regulates MutL’s clamp cycling and partner interactions can be better framed in the context of the broader GHKL family. In this family, ATP binding and hydrolysis serve as molecular switches that trigger conformational transitions, licensing active states, and resetting them for subsequent rounds. GHKL proteins are unified by a Bergerat-fold ATP-binding motif, whose hallmark “lid” region opens and closes to regulate nucleotide access and product release, linking nucleotide state to downstream activity (reviewed in [27,28]) (Figure 2C).

Despite carrying out diverse cellular functions, including DNA topology control, protein folding, signal transduction, and MMR, GHKL proteins share the strategy of ATP-dependent clamp cycling. Structural variations in the ATP-binding pocket and lid control how each member translates nucleotide binding and hydrolysis into distinct outputs, with ATP binding generally driving large conformational rearrangements that enable dimerization, higher-order assembly, and activity [39,40,45,56,64,66,67]. These ATP-dependent transitions allow GHKL proteins to propagate conformational states across protein complexes and coordinate pathway-specific events, which may provide a useful framework for understanding how MutLα uses ATP in MMR.

## 3. Mechanistic Insights from Other GHKL Family Members

### 3.1. DNA Gyrase B

**Figure 3:**
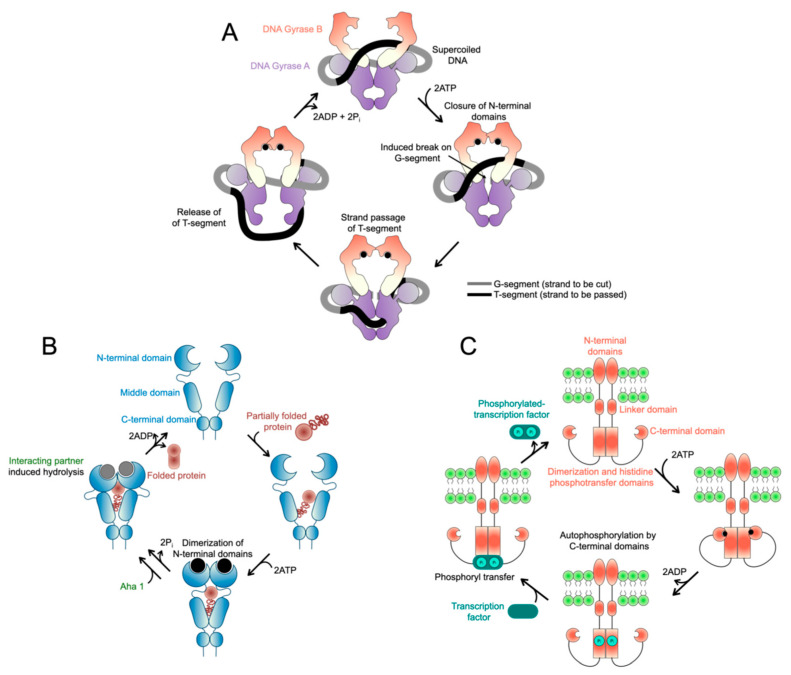
ATPase cycles of DNA gyrase, Hsp90, and histidine kinase. (**A**) DNA gyrase. DNA gyrase A subunits (GyrA) bind supercoiled DNA, with the G-segment (grey) positioned for cleavage and the T-segment (black) destined for strand passage. DNA gyrase B (GyrB) binds two molecules of ATP (black circles), driving dimerization of its N-terminal domains and concerted cleavage of the G-segment by GyrA [73,94]. The T-segment is then passed through the double-strand break, released, and the enzyme resets as ATP hydrolysis opens the GyrB N-terminal domains for another catalytic cycle. Adapted from model in [84]. (**B**) Hsp90. The N-terminal domains of the homodimer bind ATP following engagement of a client polypeptide (brown), leading to dimerization and formation of a closed state. Co-chaperones such as Aha1 (green) interact with the middle and N-terminal domain to stimulate ATP hydrolysis, releasing inorganic phosphate while ADP (grey circles) remains bound. The folded client and ADP are subsequently released, resetting the chaperone. Adapted from models in [95,96]. (**C**) Histidine kinase. Membrane phospholipids are depicted in green. ATP binding promotes interactions between the C-terminal ATPase domains and the phosphotransfer domains, leading to autophosphorylation of a conserved histidine. Hydrolysis and phosphotransfer then relay the phosphoryl group to a response regulator, typically a transcription factor (teal). Phosphoryl transfer resets the kinase for another round of sensing and signaling. Adapted from models in [97,98].

Among GHKL proteins, DNA gyrase B (GyrB) is the most extensively studied and provides a well-defined example for how ATP hydrolysis can coordinate large conformational changes with catalytic turnover. GyrB is a subunit of bacterial DNA gyrase, a type II topoisomerase composed of two DNA gyrase A (GyrA) and two GyrB subunits [73,74]. During replication and transcription, torsional strain ahead of the replication fork generates positive supercoiling that can inhibit processivity [75,76]. DNA gyrase relieves this strain through a strand-passage mechanism involving transient cleavage of one DNA duplex (the G-segment), passage of a second duplex (the T-segment) through the break, and resealing. This introduces negative supercoils and maintains topological homeostasis [77,78] (Figure 3A).

ATP binding and hydrolysis by GyrB drive a conformational cycle that closely parallels MutL and MutLα’s clamp transitions. DNA is first bound by the GyrA subunit; upon binding two ATP molecules, the N-terminal domains of GyrB dimerize, locking the enzyme into a closed state competent for strand passage [79,80]. A double-strand break is introduced into the G-segment, the T-segment is passed through, and the linking number of the DNA is altered [81,82,83,84]. After passage, the T-segment is released, and ATP hydrolysis at the GyrB N-terminal domains resets the enzyme for another round of catalysis. Biochemical studies support this model. FRET and proteolysis assays show that *apo* GyrB is an open clamp, ATP binding induces compaction into a closed state essential for DNA capture, and structural analyses confirm these transitions [79,84,85,86,87,88]. Pull-down assays with using ATP hydrolysis mutants further demonstrate that dimerization is strictly ATP-dependent [80]. Once ATP is bound, the B-gate closes to capture the T-segment, and subsequent hydrolysis reopens the gate for recycling [89,90,91].

Substrate length also influences the cycle, highlighting another parallel with MutL proteins. Longer DNA fragments stimulate more efficient ATP hydrolysis [92]. MutL homologs likewise bind more stably to extended substrates and show enhanced endonuclease activity on long DNA [57,59,60]. The underlying reasons for this similarity may differ between proteins, though. For DNA gyrase, increased flexibility of longer DNA could enhance strand passage, whereas for MutL homologs, enhanced activity may involve multiple complexes bound on the same substrate or direct manipulation of DNA, for which there is recent evidence [59,61,93]. But, in both cases, ATP-driven conformational changes are regulated by DNA length, ensuring that enzymatic activity is coordinated with substrate context.

Together, these data suggest that ATP acts as a signal in GyrB where binding ATP licenses DNA capture by the gyrase, while hydrolysis resets the enzyme for successive rounds. This “capture-and-reset” cycle offers a compelling parallel to MutL and MutLα’s clamp-like dynamics, raising the possibility that ATP hydrolysis plays a similar role in resetting DNA-bound MutL and MutLα complexes during MMR.

### 3.2. Heat Shock Protein 90

Heat shock protein 90 (Hsp90) illustrates how the GHKL ATPase fold can be adapted to protein quality control rather than DNA transactions, yet it still deploys ATP in a way that may mirror MutL and MutLα’s activities. Hsp90 is a highly conserved molecular chaperone present in bacteria and eukaryotes, where it promotes folding of a wide range of client proteins under both basal and stress conditions [99,100,101,102]. Proper Hsp90 activity is essential for cell viability. Polypeptide clients that fail to engage productively are often degraded, leading to loss of critical cellular functions, and interactome studies underscore its role as a central hub that links ATP-driven conformational dynamics to proteome stability [103,104]. Hsp90 functions as a homodimer, with each monomer containing three domains: an N-terminal ATPase domain, a middle domain that binds clients, and a C-terminal dimerization domain [105,106,107].

ATP binding and hydrolysis drive large conformational transitions between open and closed states (Figure 3B). In the *apo* form, Hsp90 remains open, where it can bind to a client protein [96,108]. After client binding, Hsp90 will bind ATP inducing a closed conformation, with dimerized N-terminal domains that license client folding [99,106,108,109,110,111,112]. ATP hydrolysis acts as a molecular switch that primes Hsp90 for client protein release [96,113,114]. Following ATP hydrolysis, the N-terminal domains remain bound to ADP. The conformation of this ADP-bound state remains debated: some studies suggest ADP stabilizes a compact form, while others propose it represents an intermediate between the *apo* and fully closed states [110,115,116]. Nonetheless, the consensus is that the ADP-bound state promotes client release and the chaperone resets by reopening the dimer upon ADP release. Thus, ATP establishes the active state, while hydrolysis defines the reset step, following the same “capture-and-reset” logic observed in GyrB.

This ATP-driven cycle is strongly modulated by co-chaperones. Aha1, one of the best studied, stimulates ATPase activity by inducing conformational changes that accelerate dimer closure and thereby enhance client processing [117,118,119,120]. Functional studies using denatured firefly luciferase underscored the importance of this regulation. Here, inhibitors disrupting the Aha1-Hsp90 interaction reduced ATPase activity and impaired refolding capacity, directly linking nucleotide turnover to enzymatic function [118]. Enzymatic cycling between ATP and ADP in the binding site thus serves as a molecular signal that couples client engagement with release [115,116].

While MutL/MutLα and GyrB use ATP to regulate clamp cycles on DNA, Hsp90 similarly clamps polypeptide substrates with ATP binding allowing the protein to engage with the client, with subsequent hydrolysis resetting the chaperone. These parallels may underscore a unifying principle of the GHKL family in that ATP binding could establish an active state, while hydrolysis triggers release and reset.

### 3.3. Histidine Kinases

Histidine kinases represent the signaling branch of the GHKL family. They are primarily transmembrane proteins and form the sensory module of two-component systems, which are widespread in bacteria and also present in eukaryotes, where their mechanisms are less understood [121,122,123]. Through their N-terminal transmembrane domains, histidine kinases detect environmental changes such as nutrient availability, pH, oxygen, or light [124,125,126,127,128]. Upon stimulus detection, the cytoplasmic C-terminal domains, which harbor the Bergerat-fold, use ATP to autophosphorylate a conserved histidine [129,130]. The phosphoryl group is then transferred to a response regulator, usually a transcription factor, to modulate gene expression [131,132,133] (Figure 3C).

Histidine kinases typically function as asymmetric homodimers. Their N-terminal transmembrane helices form helical hairpins, while the cytoplasmic domains bind and hydrolyze ATP to drive phosphorylation [123,129]. Like other GHKL proteins, they undergo nucleotide-dependent conformational transitions. Notably, ion mobility-mass spectrometry of the histidine kinase ExsG, which assembles into a higher-order homohexamer rather than the canonical dimer, revealed distinct open and closed subunit states, with ATP binding favoring the open, active conformation [134]. NMR studies of light-responsive histidine kinases similarly showed that ATP stabilizes secondary structure. In that study, the authors found that when bound to ATP in an active state, the histidine kinase is structurally stabilized compared to an ADP-bound state [135]. These findings highlight the conservation of ATP-driven conformational switching across GHKLs, but also the divergence in how conformations are linked to activity. In contrast to MutL or GyrB, where the closed state is generally the productive one, histidine kinases couple their open state to signaling.

Viewed in the broader GHKL context, histidine kinases illustrate several shared principles. ATP binding promotes structural rearrangements at the dimer interface, much like how MutL/MutLα may clamp DNA or Hsp90 clamps clients. ATP cycles also license and reset activity, where binding enables autophosphorylation, and hydrolysis eventually returns the kinase to a sensing-competent state. What distinguishes histidine kinases is how these transitions are wired directly into signaling. Here, ATP binding not only drives conformational change but also enables transfer of the phosphoryl group as the signal itself, whereas in MutL proteins, GyrB, and Hsp90, ATP hydrolysis transmits conformational signals within protein-DNA or protein-client assemblies. In all cases, the Bergerat-fold found in GHKL proteins converts ATP binding and hydrolysis into a communication mechanism, adapted to the demands of each biological system.

## 4. MutLα in MMR: Old Puzzles, New Views

Despite decades of study, MutL/MutLα’s ATPase cycle and how it contributes to efficient MMR remains incompletely understood. Mutational studies leave no doubt that ATP is required, but the central mechanistic questions persist: does ATP binding primarily act as a licensing step that enables clamp closure and partner engagement, does hydrolysis function as a reset mechanism that disengages MutL/MutLα from DNA, or do both steps cooperate in a coordinated cycle? As outlined in Section 1, structural and functional studies accentuate these questions rather than resolve them, highlighting subunit asymmetry, higher-order assemblies, and context-dependent roles for ATP. Viewed through the broader GHKL paradigm, these features suggest that MutL proteins may use ATP at multiple points, both to establish active states and to reset them, making ATP hydrolysis a potential recurring signal rather than a single trigger in MMR.

Although GHKL family members share ATP-dependent clamp dynamics, their activation mechanisms have diverged to suit their biological roles. For example, Hsp90 relies on co-chaperones such as Aha1 to stimulate lid closure and accelerate ATP turnover, whereas DNA gyrase couples ATP binding and hydrolysis directly to DNA strand passage through a series of coordinated gate movements. MutLα diverges from both systems in an additional way: it is a heterodimer in which both subunits are GHKL ATPases with Bergerat folds, yet they bind and hydrolyze ATP asymmetrically and engage distinct regulatory partners. In particular, PCNA interacts specifically with the PMS2/Pms1 subunit, stimulating MutLα’s endonuclease activity [70] and likely contributing to a more complex and regulated ATPase cycle than is observed for other GHKL enzymes. These distinctions illustrate how the conserved Bergerat-fold ATPase switch may be adapted to particular pathways and motivate our proposed model in which MutLα undergoes a unique, asymmetric, less-concerted, stepwise closure mechanism unlike the more concerted transitions typical of other GHKL ATPases.

Like other GHKL proteins, MutL homologs may use ATP as a licensing signal that activates their functions only in the proper context. In many family members, ATP binding is induced by specific contexts, such as DNA, partner proteins, or environmental signals, ensuring that activity is engaged only when appropriate. MutL may follow a similar logic, where its latent activities remain suppressed until interactions with DNA or downstream repair factors stimulate ATP binding, with subsequent hydrolysis promoting recycling on DNA. Viewed through this GHKL framework, past biochemical, structural, and biophysical data suggest a model in which eukaryotic MutLα employs ATP both to license activity and to drive turnover during MMR.

**Figure 4:**
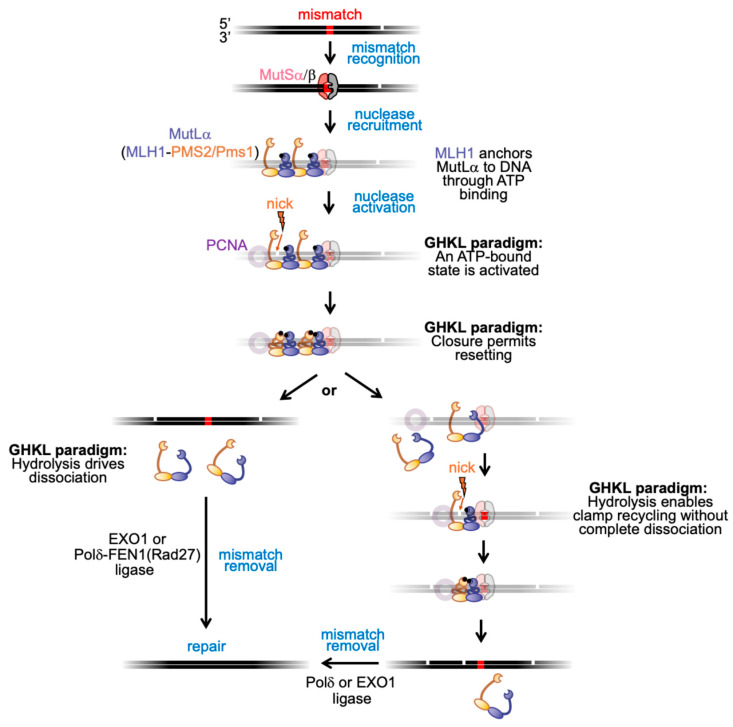
Model for MutLα’s ATPase roles in eukaryotic MMR through the GHKL paradigm. Mismatch recognition by MutSα/β triggers MutLα recruitment (MutS activities not shown in detail). To highlight MutLα ATPase clamp dynamics, MutS and DNA are greyed. MutLα ATPase is stimulated by DNA, with MLH1 (blue) positioned as the MutS-interacting anchor and PMS2/Pms1 (yellow) interacting with PCNA (purple, greyed). PCNA stimulates both endonuclease (lightning bolt) and ATPase activities. In the GHKL paradigm, ATP hydrolysis resets the clamp, leading either to iterative nicking without dissociation or to complete dissociation and recycling, which could result in iterative nicking if the pathway restarts. Mismatches can be removed by Polδ or EXO1.

Structural and biochemical studies indicate that following mismatch recognition by MutSα or MutSβ, MutLα is recruited through contacts mediated by the MLH1 subunit [136,137,138]. There are conflicting models for whether this recruitment occurs directly at the mismatch site or away from it through sliding-clamp activity [3,50,61,69,139,140,141,142,143]. This recruitment, together with the asymmetric ATPase properties of the heterodimer (discussed in Section 1) and evidence that DNA binding stimulates ATP turnover, suggests a model in which MLH1 may serve as an anchor for the initiation complex (Figure 4). In such a model, ATP binding by MLH1 may stabilize MutLα on DNA, while PMS2/Pms1 remains poised to engage activating partners, such as PCNA. It should be noted that this represents a departure from other GHKL family ATPases, where subunits typically bind ATP and undergo clamp closure in a concerted manner. In contrast, MutLα has been suggested to have two ATP-bound conformations: an asymmetric state in which only the MLH1 subunit is ATP-bound (as indicated by kinetic and AFM data [5,45]) and a fully ATP-bound state in which both subunits bind ATP and the two Bergerat folds dimerize to close the clamp. The asymmetric, MLH1 ATP-bound state may therefore constitute the active intermediate that engages partner proteins, while full closure upon ATP binding to both subunits still aligns MutLα with the broader GHKL paradigm in which ATP-bound conformations enable functional activation.

Upon interactions with PCNA, MutLα’s endonuclease activity is stimulated through interactions between the PMS2/Pms1 subunit and PCNA [25,26,64,69,70]. In minimal biochemical systems containing MutLα, PCNA, and DNA, ATP is not required for nonspecific nicking [25,26]. However, it has not been explicitly tested whether ATP plays a more stimulatory or regulatory role in fully reconstituted systems. Following endonuclease activation, PMS2/Pms1 may itself bind ATP, consistent with evidence that PCNA stimulates MutLα ATPase activity. This ATP binding could act as a signal for enzymatic reset, paralleling other GHKL proteins in which ATP binding licenses catalytic activity and hydrolysis or nucleotide release resets the complex for another cycle.

The fate of MutLα after ATP hydrolysis remains unresolved. One model proposes that hydrolysis opens the clamp and dissociates the N-terminal domains, leading to complete release of the protein from DNA. In this case, MutLα leaves behind a nick as an entry point for excision or strand-displacement synthesis, or potentially recycles to the same mismatch site to introduce additional nicks that amplify the repair signal [22,144,145,146]. An alternative model may involve iterative nicking when multiple MutLα complexes are assembled on DNA. In this model, hydrolysis-induced resetting could release the protein that performed the incision while other MutLα proteins remain bound and become primed to nick again or all members of the complex recycle but remain bound to the DNA. This is at least partially supported by DNA binding assays with yeast proteins where ternary complexes putatively consisting of MutSα, MutLα, and PCNA persist on substrates with streptavidin-bumpered ends in the presence of ATP and magnesium [69]. A related possibility is that MutLα, alone or in complex with MutSα/β exploits sliding-clamp properties to reset without full dissociation, thereby facilitating repeated rounds of incision. Such a mechanism echoes broader themes across the GHKL family, where ATP functions not only as a license for activity but also as a reset signal that drives iterative, context-dependent cycles of function. At present, however, these models remain speculative, and more explicit experimental tests will be required to distinguish among them. It will also be important to determine whether different eukaryotic MutLα proteins vary in how they utilize ATP, and how these modes compare with bacterial MutL homologs and their subunits.

## 5. Broader Implications and Future Directions

Beyond the role of MutLα in canonical MMR, other MutL complexes also display ATP-dependent activities whose mechanistic basis remains unresolved. For example, genetic studies highlight the importance of ATP for MutLγ during meiosis, yet biochemical work shows that MutLγ can be activated on DNA junctions even in the absence of nucleotide [147,148]. This raises the question of whether ATP plays an analogous role for MutLγ as it does for MutLα. MutLβ is even less well understood, with its biological role in MMR and meiosis only beginning to be elucidated [32,35,149,150,151,152], but its conservation across eukaryotes suggests that ATPase activity may contribute to functions not yet fully defined. Considering these gaps through the broader GHKL framework provides a way to generate new hypotheses about how distinct MutL complexes adapt ATP usage to their specialized contexts.

More broadly, lessons from MMR can inform our understanding of other GHKL proteins beyond MutL homologs. Several GHKL ATPases act on chromatin: microrchidia (MORC) proteins use their ATPase domains in chromatin remodeling and gene regulation [153,154,155,156], and SMCHD1, which has also been proposed to adopt the shared GHKL ATPase fold, may employ clamp-like dynamics to reshape chromatin architecture [157,158,159]. Similarly, topoisomerase VI subunits such as the meiotic factor TOP6BPL retain the conserved GHKL lid and may couple ATP hydrolysis to conformational transitions and DNA processing in ways reminiscent of MutL [160]. Recent structural work has further revealed that the neurodegenerative disease-linked protein sacsin contains a Bergerat-fold very similar to Hsp90 proteins, which may suggest chaperone-like activities [161]. By explicitly comparing across this family, we can uncover both shared mechanistic strategies and unique adaptations that clarify a wide spectrum of biological pathways.

Advances in structural and computational tools now make it possible to probe these questions with unprecedented resolution. Cryo-EM and single-molecule approaches can directly visualize conformational states and DNA transactions, while deep-learning-based structure prediction platforms such as AlphaFold offer new opportunities to model elusive intermediates and higher-order assemblies. Integrating these approaches with classical genetics and biochemistry will be key not only to defining how ATP hydrolysis is wired into the diverse activities of known GHKL proteins, but also to uncovering conserved activities that may guide the identification of new family members and unanticipated biological roles.

## Data Availability

No new data were created or analyzed in this study. Data sharing is not applicable to this article.
